# Comparative Efficacy and Safety of Tirzepatide in Asian and Non‐Asian Adults With Obesity Without Diabetes: A Systematic Review and Meta‐Analysis

**DOI:** 10.1002/edm2.70291

**Published:** 2026-07-22

**Authors:** A. B. M. Kamrul‐Hasan, Ibrahim Khalil, Deep Dutta, Kunal Mahajan, Lakshmi Nagendra, Joseph M. Pappachan

**Affiliations:** ^1^ Department of Endocrinology Mymensingh Medical College Mymensingh Bangladesh; ^2^ Department of Medicine Dhaka Medical College and Hospital Dhaka Bangladesh; ^3^ Department of Endocrinology CEDAR Superspeciality Healthcare Dwarka New Delhi India; ^4^ Department of Cardiology Himachal Heart Institute Mandi Himachal India; ^5^ Department of Endocrinology JSS Medical College, JSS Academy of Higher Education and Research Mysore India; ^6^ Department of Endocrinology and Metabolism Lancashire Teaching Hospitals NHS Trust Preston UK; ^7^ Faculty of Science Manchester Metropolitan University Manchester UK; ^8^ Department of Endocrinology, Kasturba Medical College Manipal Academy of Higher Education Manipal India

**Keywords:** adverse events, Asian, non‐Asian, obesity, tirzepatide, weight loss

## Abstract

**Introduction:**

Tirzepatide, a dual GIP/GLP‐1 receptor agonist, produces substantial weight loss in adults with obesity. Given ethnic differences in obesity phenotype and cardiometabolic risk, this systematic review and meta‐analysis compared tirzepatide's efficacy and safety between Asian and non‐Asian adults without diabetes.

**Methods:**

This PROSPERO‐registered review (CRD420261361351) searched PubMed, Scopus, Web of Science and ClinicalTrials.gov through 15 March 2026. Phase 3 SURMOUNT randomised, placebo‐controlled trials were classified as Asian (100% Asian participants) or predominantly non‐Asian (< 25% Asian participants). The primary outcome was the percentage change in body weight. Secondary outcomes included absolute weight change, body mass index (BMI), waist circumference, weight loss thresholds (≥ 5%, ≥ 10%, ≥ 15%) and adverse events. Random‐effects meta‐analyses and meta‐regression were performed.

**Results:**

Five trials (six comparisons; *N* = 4022) were included: two Asian (SURMOUNT‐CN, SURMOUNT‐J; *n* = 148 tirzepatide‐treated) and four predominantly non‐Asian. Tirzepatide 15 mg/maximum tolerated dose produced an 18.04% greater reduction in body weight versus placebo (95% CI −19.86 to −16.22; *p* < 0.00001, *I* ^2^ = 77%). Percentage weight loss appeared comparable between Asian (−18.16%) and non‐Asian (−17.92%) trials (*p* = 0.94), but this underpowered comparison cannot confirm equivalence. Absolute weight loss was greater in non‐Asians (−23.46 vs. −16.72 kg; *p* = 0.03), largely reflecting higher baseline weight. Safety profiles were similar, with no statistically significant interactions by ethnic subgroup.

**Conclusions:**

Tirzepatide provides clinically meaningful weight reduction, with broadly similar relative percentage efficacy across Asian and non‐Asian trial populations. These exploratory, indirect findings do not establish ethnic equivalence and require confirmation in adequately powered, ethnicity‐stratified trials.

**Registration:**

The meta‐analysis was registered in PROSPERO under the registration number CRD420261361351. The review protocol summary is available on the PROSPERO website.

## Graphical abstract

Tirzepatide 15 mg produces large, clinically meaningful weight loss in adults with obesity without diabetes, with similar percentage weight loss in Asian (−18.16%) and predominantly non‐Asian (−17.92%) trials and comparable safety, though ethnic comparisons remain exploratory and underpowered for definitive conclusions.

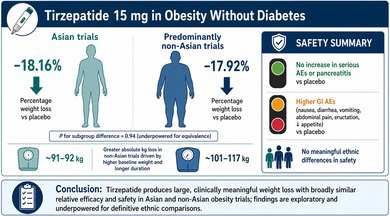

**Abbreviations**
AEs: adverse events
BMI: body mass index
CI: confidence interval
MD: mean difference
MTD: maximum tolerated dose
NA: not applicable
PRISMA: Preferred Reporting Items for Systematic Reviews and Meta‐Analyses
RCT: randomised controlled trials
RevMan: Review Manager
RoB: risk of bias
RR: risk ratio
SD: standard deviation
SR/MA: systematic review and meta‐analysis
WC: waist circumference

## 1 Introduction

Obesity without diabetes is a major and growing public health challenge worldwide, contributing substantially to cardiovascular disease, metabolic dysfunction, certain cancers, impaired physical function and premature mortality. Contemporary obesity care increasingly recognises obesity as a chronic, relapsing disease that requires long‐term management rather than a short‐term weight‐loss intervention alone. Accordingly, optimal treatment extends beyond calorie restriction to a multimodal framework that incorporates nutritional therapy, physical activity, behavioural support, pharmacotherapy, and, when indicated, metabolic‐bariatric surgery [1].

These principles are particularly relevant in Asian populations, where obesity‐related cardiometabolic risk often occurs at lower body mass index (BMI) levels than in Western populations because of a greater tendency towards visceral and ectopic fat accumulation at a given BMI [2]. Risk may also vary across Asian countries and among ethnic groups, even among people living in similar environments within the same country. On the basis of epidemiologic studies and expert consensus, the WHO Expert Consultation and regional guidelines for South and Southeast Asia recommend lower BMI and waist‐circumference thresholds for overweight and obesity in Asian populations, reflecting higher cardiometabolic risk at a given BMI [3, 4, 5]. In general, these guidelines use BMI ≥ 23 kg/m^2^ for overweight and ≥ 25 kg/m^2^ or ≥ 27.5 kg/m^2^ for obesity, together with lower waist circumference thresholds than in Western populations [3, 4, 5, 6]. In addition, recent South and Southeast Asian consensus recommendations emphasise that obesity management should be culturally contextualised, incorporating locally available foods, culinary patterns, family structure, stigma, affordability and healthcare‐system constraints, while still adhering to the core pillars of evidence‐based chronic disease care [6].

Pharmacotherapy for obesity, often referred to as obesity management medications (OMMs), is becoming an increasingly important component of obesity management when lifestyle intervention alone is insufficient, especially for people with obesity‐related complications or high‐risk adiposity phenotypes [7]. Tirzepatide, a once‐weekly dual glucose‐dependent insulinotropic polypeptide (GIP) and glucagon‐like peptide‐1 (GLP‐1) receptor agonist, has emerged as one of the most effective anti‐obesity agents currently available, producing substantial and dose‐dependent reductions in body weight alongside improvements in several cardiometabolic risk factors [8]. However, it is clinically important to determine whether the efficacy and tolerability of tirzepatide are similar between Asian and non‐Asian adults, as factors such as baseline body composition, BMI‐based treatment criteria, dietary habits and tolerability profiles may vary across populations.

The SURMOUNT clinical‐trial programme comprises a series of phase 3 randomised trials evaluating once‐weekly tirzepatide versus placebo (and, in some cases, active comparators) in adults with obesity or overweight and weight‐related complications, with or without diabetes. It includes multinational trials as well as trials conducted exclusively in China and Japan, providing a coherent evidence base to assess tirzepatide's efficacy and safety in Asian and predominantly non‐Asian populations with obesity without diabetes [9, 10, 11, 12, 13]. Without randomised controlled trials (RCTs) by ethnicity, a systematic review and meta‐analysis (SR/MA) of existing trial‐level data offers the most current, though indirect, evidence on potential similarities and differences in tirzepatide's efficacy and safety among Asian and non‐Asian adults with obesity but without diabetes. Accordingly, this systematic review and meta‐analysis aimed to address the following question: among adults with obesity without diabetes enrolled in the phase 3 SURMOUNT trials, are tirzepatide's efficacy and safety profiles similar between Asian and predominantly non‐Asian populations? Secondary aims were to describe absolute and relative weight loss, adiposity indices and adverse events by ethnic trial group and to explore trial‐level moderators of these outcomes.

## 2 Methods

### 2.1 Study Registration and Reporting

This systematic review and meta‐analysis was registered with PROSPERO (CRD420261361351) and is reported in accordance with the PRISMA 2020 checklist and the Cochrane Handbook for Systematic Reviews of Interventions [14, 15].

### 2.2 Search Strategy

A systematic literature search was conducted in PubMed, Scopus, Web of Science and ClinicalTrials.gov from database inception to 15 March 2026. Search terms included ‘tirzepatide,’ ‘LY3298176,’ ‘obesity,’ and ‘overweight,’ combined with Boolean operators to find recent or unpublished clinical trials in any language. Detailed search strings for each database are provided in Table S1. Reference lists of included trials and relevant systematic reviews were hand‐searched to identify additional studies.

### 2.3 Eligibility Criteria

The Population, Intervention, Comparison, Outcomes and Study (PICOS) framework guided the development of eligibility criteria for the RCTs included in this SR/MA. The patient population (P) comprised adults with obesity or overweight and weight‐related comorbidities but without diabetes. The intervention (I) was once‐weekly subcutaneous tirzepatide. The control (C) group received either a placebo or an active weight‐loss intervention. The outcomes (O) included changes in body weight (percent and absolute) from baseline and adverse events (AEs) associated with the interventions. Only RCTs (S) with a minimum duration of 12 weeks were included. Excluded were animal studies, phase 1 trials, non‐randomised designs, retrospective analyses, pooled post hoc analyses, conference abstracts, letters to the editor and case reports.

### 2.4 Trial Classification

Included trials were classified as Asian if 100% of participants were enrolled from Asian countries (SURMOUNT‐CN: China; SURMOUNT‐J: Japan) [11, 12]. Trials were classified as predominantly non‐Asian if fewer than 25% of enrolled participants were of Asian ethnicity (SURMOUNT‐1: 10.9% Asian; SURMOUNT‐3: 0.7% Asian; SURMOUNT‐OSA(1): 20.1% Asian; SURMOUNT‐OSA(2): 14.1% Asian) [9, 10, 13]. This threshold was chosen to maximise ethnic contrast between groups, conceptually aligned with prior ethnicity‐focused tirzepatide analyses in type 2 diabetes that compared Asian and non‐Asian subgroups using trial‐level ethnic composition, while recognising that none of these analyses involved randomisation by ethnicity [16]. SURMOUNT‐OSA(1) and SURMOUNT‐OSA(2) enrolled adults with obesity and moderate‐to‐severe OSA and are therefore considered obesity trials with a complication. Because ethnicity‐stratified efficacy and safety data were not reported, both trials were classified as predominantly non‐Asian (20.1% and 14.1% Asian participants, respectively), recognising that this may introduce some ethnic misclassification [13]. It is essential to note that no SURMOUNT trial randomised participants by ethnicity. Therefore, the Asian versus non‐Asian comparison in this meta‐analysis is an indirect, ecological‐level analysis. Observed differences (or their absence) between subgroups cannot be causally attributed to ethnicity per se and may reflect between‐trial confounding differences in study populations, eligibility criteria, dosing regimens, cultural dietary patterns and geographic settings. All conclusions should be interpreted in light of this inferential limitation.

### 2.5 Outcomes

The primary outcome of this SR/MA was the percentage change in body weight from baseline. Secondary outcomes included absolute changes in body weight, BMI and waist circumference (WC), as well as the proportions of subjects achieving weight reductions of ≥ 5%, ≥ 10% and ≥ 15%. Adverse events (AEs), particularly gastrointestinal (GI) issues, were also assessed.

### 2.6 Data Extraction and Dealing With Missing Data

Two authors independently extracted data using standardised forms. When multiple publications from the same study group were identified, results were combined and relevant data from each report were included in the analysis. Data on primary and secondary outcomes, as previously mentioned, were collected. Patient characteristics, including demographic details, were recorded in a table. Disagreements were adjudicated by a third author. The Supporting Information of the relevant studies were thoroughly reviewed. Any pertinent information was obtained via email correspondence with the corresponding author and thoughtfully integrated into the meta‐analysis. A detailed review of key numerical data, including the numbers of individuals screened and randomised and a careful examination of the intention‐to‐treat, as‐treated and per‐protocol populations were conducted. Additionally, attrition rates, including dropouts, losses to follow‐up and withdrawals, were carefully assessed.

### 2.7 Risk of Bias Assessment

Two authors independently conducted the risk of bias (RoB) assessment using version 2 of the Cochrane risk‐of‐bias tool for randomised trials (RoB 2) [17]. The domains covered by RoB 2 include all recognised types of bias that can affect RCT results, such as bias from the randomisation process, bias due to deviations from intended interventions, bias caused by missing outcome data, bias in outcome measurement and bias in the selection of reported results. The RoB judgement classified each domain into one of three levels: low RoB, some concerns or high RoB. The overall RoB for each result was determined by the least favourable assessment across the bias domains [17]. Disagreements were adjudicated by a third author.

### 2.8 Statistical Analysis

Forest plots and meta‐analyses were performed using RevMan Web (version 10.5.0; Cochrane Collaboration, 2026) [18]. Results are expressed as mean differences (MDs) for continuous outcomes and risk ratios (RRs) for dichotomous outcomes, with 95% confidence intervals (CIs). Random‐effects models with inverse‐variance weighting were applied throughout to account for expected between‐trial heterogeneity in study populations and durations. Heterogeneity was quantified using the *I* ^2^ statistic and assessed using the chi‐square test; *I* ^2^ values of 25%, 50% and 75% were considered low, moderate and high, respectively [19]. Subgroup (Asian versus non‐Asian) comparisons were performed using the chi‐square test for subgroup interaction. Meta‐analyses were conducted when outcome data from at least two trials were available for each Asian and non‐Asian group. A significance level of *p* < 0.05 was used. Trial‐level mean baseline body weight, BMI, age, percentage of female participants and trial duration were used as continuous moderators in univariable meta‐regression for the percentage and absolute weight change, using the method of moments estimator in RStudio (Version 2026.01.1+403) [20]. Given the very limited number of trials, these analyses are exploratory. A pre‐specified sensitivity analysis was conducted, restricting comparisons to trials with fixed‐dose tirzepatide 15 mg arms only (SURMOUNT‐1, SURMOUNT‐CN, SURMOUNT‐J), to evaluate the impact of including MTD arms on the percentage and absolute changes in body weight.

## 3 Results

### 3.1 Study Selection

**FIGURE 1:**
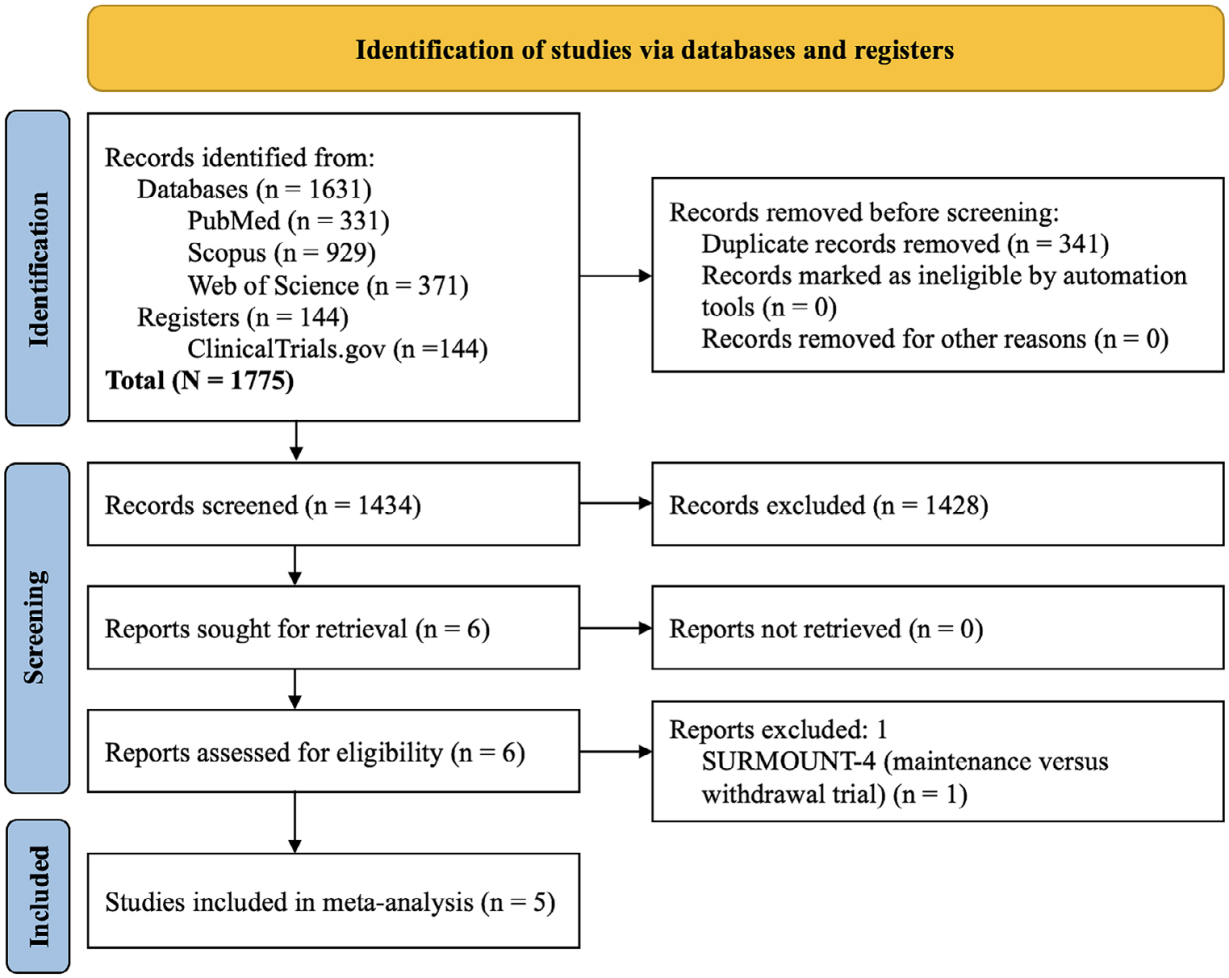
Flowchart on study retrieval and inclusion in the meta‐analysis.

Figure 1 illustrates the study selection process. A total of 1775 articles were identified; after screening, five RCTs (six treatment comparisons; *N* = 4022) were included [9, 10, 11, 12, 13]. SURMOUNT‐4 was excluded because it evaluated maintenance versus withdrawal of tirzepatide rather than weight‐loss induction [21].

### 3.2 Characteristics of Included Trials

All included RCTs were phase 3, double‐blind, placebo‐controlled and lasted 52–72 weeks. SURMOUNT‐OSA consisted of two separate RCTs, analysed independently in this SR/MA as SURMOUNT‐OSA(1) and SURMOUNT‐OSA(2) [13]. Thus, two trials were classified as Asian (SURMOUNT‐CN: 52 weeks, *n* = 140; SURMOUNT‐J: 72 weeks, *n* = 152) [11, 12] and four as predominantly non‐Asian (SURMOUNT‐1: 72 weeks; SURMOUNT‐3: 72 weeks; SURMOUNT‐OSA(1) and OSA(2): 52 weeks each) [9, 10, 13]. Key baseline characteristics are summarised in Table 1. Mean baseline body weight was substantially lower in Asian trials (approximately 91–92 kg) than in non‐Asian trials (approximately 101–117 kg), and baseline BMI was lower in Asian trials (32–34 kg/m^2^) compared to non‐Asian trials (36–40 kg/m^2^).

**TABLE 1:** The basic and baseline characteristics of the included randomised controlled trials and participants.

Trial name [ref.], clinical trial identifier, authors, publication year, place of study	Major baseline characteristics of the study subjects	Study arms	*N*	Age (years), mean ± SD	Female (%)	Body weight (kg), mean ± SD	BMI (kg/m^2^), mean ± SD	WC (cm), mean ± SD	Duration
SURMOUNT‐1 [9], NCT04184622, Jastreboff et al. 2022, Nine countries in Asia, North and South America	Adults with BMI ≥ 30 kg/m^2^ or BMI ≥ 27 kg/m^2^ with ≥ 1 weight‐related complication ≥ 1 unsuccessful dietary effort to lose weight Asian: 10.9%	Tirzepatide 5 mg QW	630	45.6 ± 12.7	67.6	102.9 ± 20.7	37.4 ± 6.6	113.2 ± 14.3	72 weeks
		Tirzepatide 10 mg QW	636	44.7 ± 12.4	67.1	105.8 ± 23.3	38.2 ± 7.0	114.8 ± 15.8	
		Tirzepatide 15 mg QW	630	44.9 ± 12.3	67.5	105.6 ± 22.9	38.1 ± 6.7	114.4 ± 15.6	
		Placebo	643	44.4 ± 12.5	67.8	104.8 ± 21.4	38.2 ± 6.9	114.0 ± 14.9	
SURMOUNT‐3 [10], NCT04657016, Wadden et al. 2023, USA, Argentina and Brazil	Adults with BMI ≥ 30 kg/m^2^ or BMI ≥ 27 kg/m^2^ with ≥ 1 weight‐related comorbidity ≥ 1 unsuccessful dietary effort to lose weight Achieved ≥ 5% weight reduction after a 12‐week intensive lifestyle intervention Asian: 0.7%	Tirzepatide MTD QW (Tirzepatide 15 mg: 86.4%)	287	45.4 ± 12.6	63.1	102.5 ± 22.1	36.1 ± 6.1	109.3 ± 15.2	72 weeks
		Placebo	292	45.7 ± 11.8	62.7	101.3 ± 20.7	35.7 ± 6.4	109.6 ± 15.1	
SURMOUNT‐CN [11], NCT05024032, Zhao et al. 2024, China	Adults with BMI ≥ 28 kg/m^2^ or BMI ≥ 24 kg/m^2^ with ≥ 1 weight‐related comorbidity ≥ 1 unsuccessful dietary effort to lose weight Asian: 100%	Tirzepatide 10 mg QW	70	34.7 ± 7.2	50.0	92.2 ± 16.2	32.6 ± 4.1	105.0 ± 10.4	52 weeks
		Tirzepatide 15 mg QW	71	35.8 ± 9.3	49.3	91.3 ± 16.2	32.0 ± 3.7	104.2 ± 10.9	
		Placebo	69	37.8 ± 10.2	47.8	92.0 ± 15.8	32.4 ± 3.6	105.3 ± 10.4	
SURMOUNT‐J [12], NCT04844918, Kadowaki et al. 2025, Japan	Age ≥ 20 years; BMI 27–35 kg/m^2^ with ≥ 2 obesity‐related comorbidity or BMI ≥ 35 kg/m^2^ with ≥ 1 obesity‐related comorbidity ≥ 1 unsuccessful dietary effort to lose weight Asian: 100%	Tirzepatide 10 mg QW	73	49.0 ± 10.9	41	92.4 ± 15.0	33.2 ± 4.1	107.7 ± 9.8	72 weeks
		Tirzepatide 15 mg QW	77	51.1 ± 10.3	42	91.7 ± 14.8	33.6 ± 4.3	107.6 ± 10.4	
		Placebo	75	52.3 ± 10.9	40	92.0 ± 15.3	33.7 ± 4.9	108.7 ± 13.0	
SURMOUNT‐OSA(1) [13], NCT05412004, Malhotra et al. 2024, Nine countries	Adults with moderate‐to‐severe obstructive sleep apnea and BMI ≥ 30 kg/m^2^ (≥ 27 in Japan) ≥ 1 unsuccessful dietary effort to lose weight Unable or unwilling to use PAP therapy Asian: 20.1%	Tirzepatide MTD QW	114	47.3 ± 11.0	31.6	116.7 ± 24.6	39.7 ± 7.3	122.6 ± 16.6	52 weeks
		Placebo	120	48.4 ± 11.9	34.2	112.8 ± 22.6	38.6 ± 6.7	119.8 ± 14.8	
SURMOUNT‐OSA(2) [13], NCT05412004, Malhotra et al. 2024, Nine countries	Adults with moderate‐to‐severe obstructive sleep apnea and BMI ≥ 30 kg/m^2^ (≥ 27 in Japan) ≥ 1 unsuccessful dietary effort to lose weight On PAP therapy for ≥ 3 months prior to visit 1 and plan to continue PAP therapy during the study Asian: 14.1%	Tirzepatide MTD QW	120	50.8 ± 10.7	27.5	115.8 ± 21.5	38.6 ± 6.1	120.7 ± 13.1	52 weeks
		Placebo	115	52.7 ± 11.3	27.8	115.1 ± 22.7	38.7 ± 6.0	121.0 ± 14.0	

Abbreviations: BMI, body mass index; MTD, maximum tolerated dose; PAP, positive airway pressure; QW, once a week; SD, standard deviation; WC, waist circumference.

The primary analysis compared tirzepatide 15 mg once weekly with placebo. In trials that used a maximum tolerated dose (MTD) strategy (SURMOUNT‐3, SURMOUNT‐OSA), MTD arms were pooled with the fixed 15‐mg arms. In SURMOUNT‐3, most (86.4%) participants ultimately reached 15 mg, making the dosing regimens functionally equivalent at the trial level [10]. In the two SURMOUNT‐OSA trials, participants were titrated to an individually determined MTD of 10 or 15 mg once weekly. Published reports do not specify the proportion of participants who remained at 10 mg versus 15 mg at week 52, limiting detailed assessment of dose–response and introducing structural asymmetry between Asian and predominantly non‐Asian trials [13]. In all trials using MTD, dose escalation depended on individual tolerability, so participants who did not reach 15 mg are likely to have systematically different efficacy and safety profiles. This tolerability‐driven escalation creates non‐random missingness and potential selection bias, which must be considered when interpreting trial‐level ethnic comparisons in this meta‐analysis. Tirzepatide 5 and 10 mg arms were excluded from the primary meta‐analysis due to insufficient trials providing data for a dose‐specific ethnic comparison (5 mg available only in SURMOUNT‐1; 10 mg available in one non‐Asian trial [SURMOUNT‐1] and two Asian trials [SURMOUNT‐CN and SURMOUNT‐J]) [9, 11, 12]. The available 10 mg descriptive data are presented in Tables S2 and S3.

### 3.3 Risk of Bias

**FIGURE 2:**
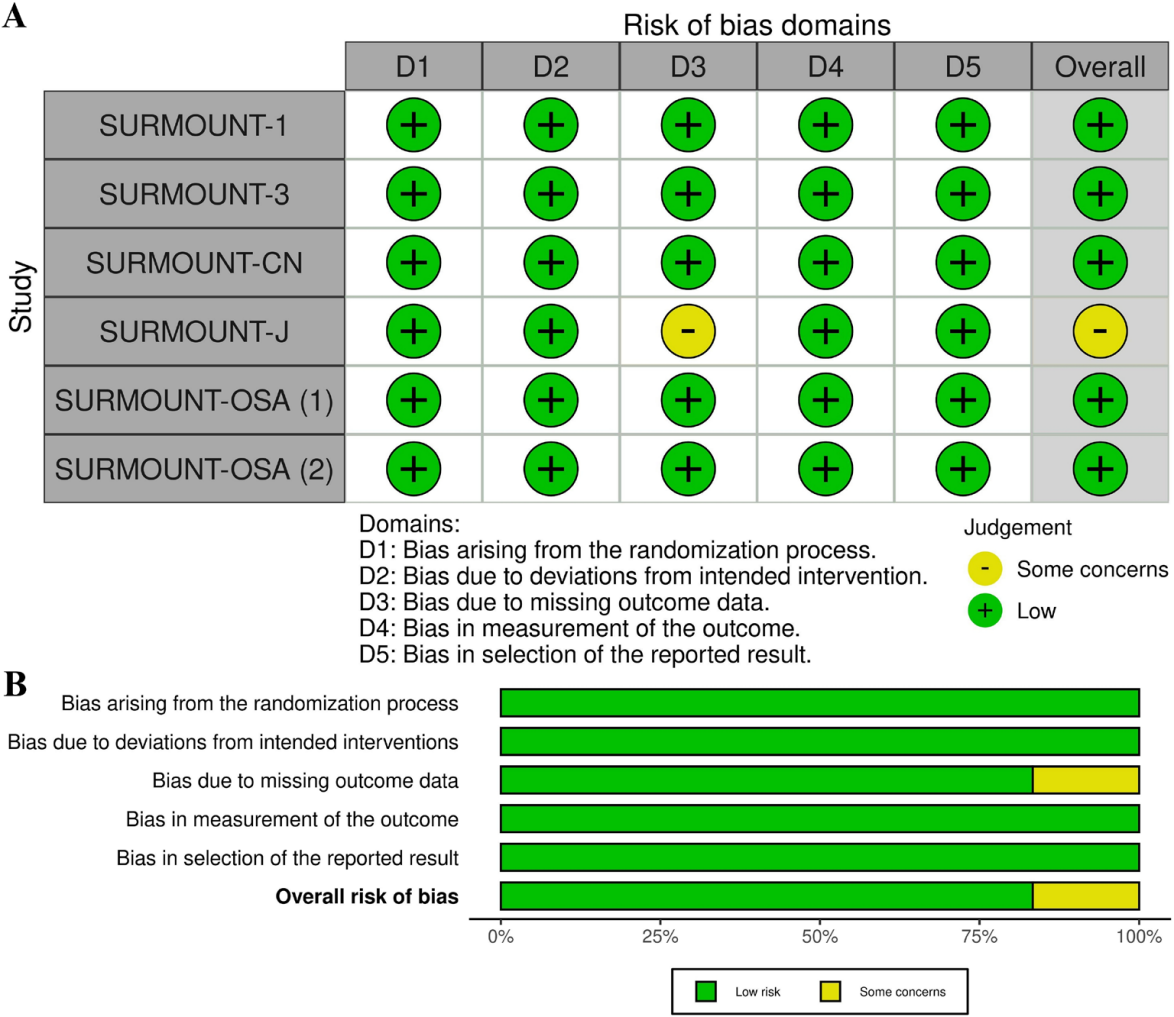
(A) Risk of bias summary: Review authors' judgements about each risk of bias item for each included study; (B) Risk of bias graph: Review authors' judgements about each risk of bias item presented as percentages across all included studies.

The risk of bias assessment indicated a low risk across most domains; however, SURMOUNT‐J raised concerns about missing outcome data (Figure 2). In SURMOUNT‐J, missing outcome data were more frequent than in other trials (15% of participants in the main analysis set discontinued study drug and 4% discontinued the study), primarily due to treatment discontinuation and loss to follow‐up; details of the handling of missing data (e.g., mixed‐model repeated measures) are described in the original report [12]. Our meta‐analysis uses published estimates and therefore inherits these trial‐level assumptions, which may contribute to uncertainty in the Asian subgroup. Publication bias was not formally evaluated using funnel plots or quantitative tests because fewer than 10 RCTs contributed to each meta‐analysis, consistent with Cochrane recommendations. Given the small number of sponsor‐funded trials, a potential risk of publication bias cannot be excluded, but any such bias remains speculative in the absence of formal assessment [15, 22].

### 3.4 Primary Outcome: Percentage Change in Body Weight

**FIGURE 3:**
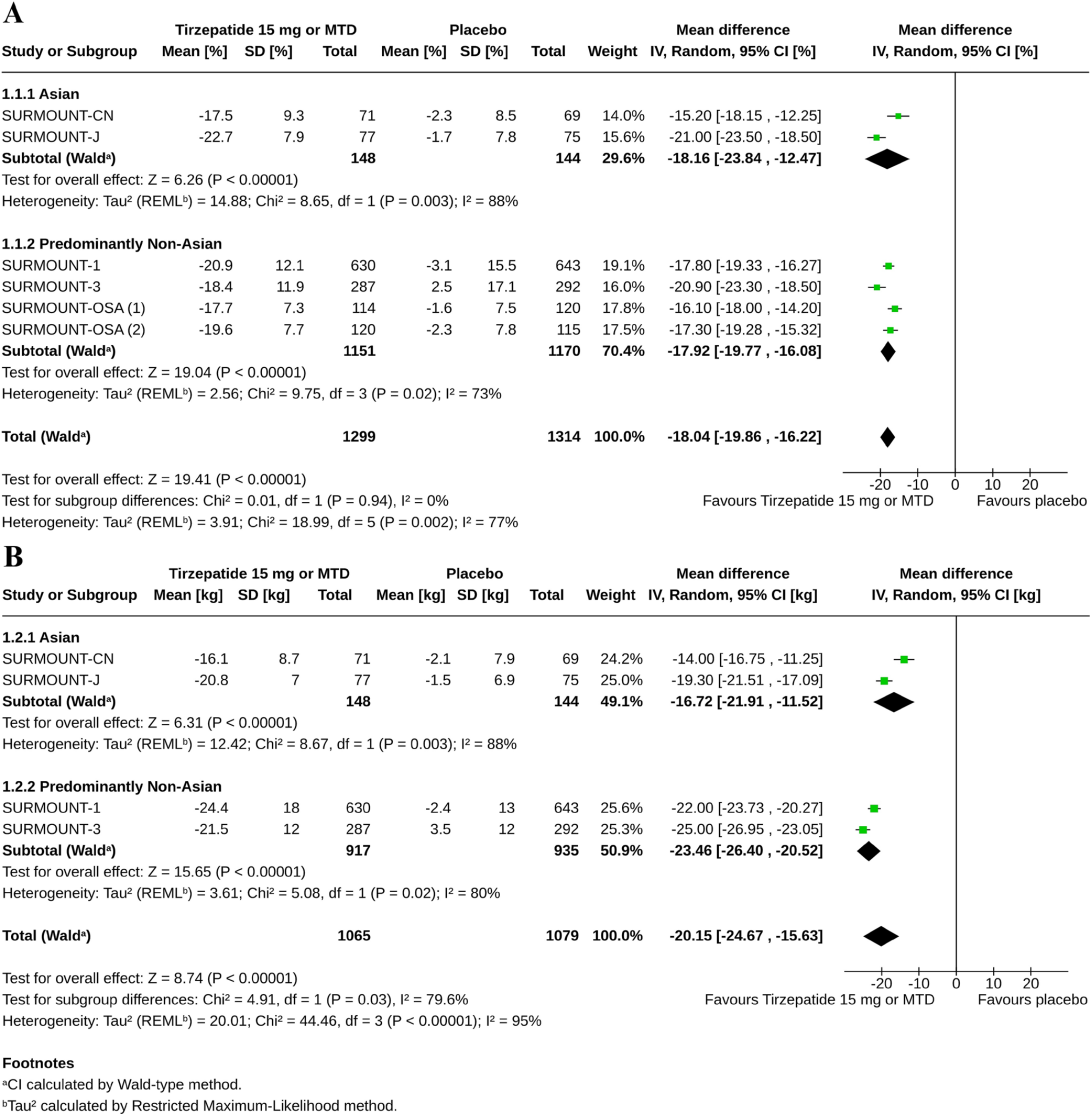
Forest plot displaying. (A) Percentage changes in body weight from baseline in the tirzepatide versus placebo groups with subgroup analyses for Asians and non‐Asians. (B) Absolute changes in body weight from baseline in the tirzepatide versus placebo groups with subgroup analyses for Asians and non‐Asians.

Tirzepatide 15 mg/MTD produced an 18.04% greater reduction in body weight versus placebo (95% CI −19.86 to −16.22; *p* < 0.00001; *I* ^2^ = 77%). Percentage weight loss was −18.16% in Asian and −17.92% in non‐Asian trials (*p* for subgroup difference = 0.94) (Figure 3A). This non‐significant subgroup test should not be interpreted as evidence of equivalence. With only two trials (*N* = 148 tirzepatide‐treated participants) in the Asian subgroup, the analysis is substantially underpowered to detect clinically meaningful between‐group differences and the result reflects an absence of statistical evidence rather than biological equivalence. Meta‐regression analyses confirmed that mean baseline body weight, BMI, age and the proportion of females were not significant moderators of the percentage weight‐loss effect (all *p* > 0.05). The only significant moderator was trial duration (*β* = −0.1643, 95% CI −0.2937 to −0.0350; *p* = 0.0128; *R* ^2^ = 64.48%), indicating that longer trials were associated with modestly greater percentage weight reduction with tirzepatide (Table S4).

### 3.5 Absolute Weight Change

Absolute weight loss was significantly greater in non‐Asians compared to Asians (−23.46 kg vs. −16.72 kg; *p* for subgroup difference = 0.03) (Figure 3B). This difference parallels the substantially higher baseline body weight in non‐Asian trials (101–117 kg vs. 91–92 kg). In contrast to the percentage change findings, absolute weight loss (kg) showed greater variability across trials. Meta‐regression analyses revealed several notable associations. Trial duration was again a significant moderator (*β* = −0.4065, 95% CI −0.7387 to −0.0744; *p* = 0.0165; *R* ^2^ = 65.58%), with longer trials associated with greater absolute weight loss. There were also trends towards greater absolute weight loss in trials with higher mean baseline BMI (*β* = −1.3824, *p* = 0.0561; *R* ^2^ = 49.48%) and higher mean baseline body weight (*β* = −0.4983, *p* = 0.0630; *R* ^2^ = 47.79%) (Table S5). These findings suggest that differences in absolute weight loss between populations are at least partly explained by higher baseline body weight and BMI in non‐Asian trials.

Given only six trial‐level comparisons for percentage and four for absolute weight change, all meta‐regression results should be considered exploratory and hypothesis‐generating. In absolute weight change analyses, higher mean baseline BMI and body weight showed borderline associations with greater kilogram loss (*p* = 0.0561 and *p* = 0.0630, respectively), suggesting a plausible influence of starting body size but falling short of conventional statistical significance.

### 3.6 Other Efficacy Outcomes

Tirzepatide 15 mg/MTD produced greater reductions in BMI (MD −7.21 kg/m^2^; 95% CI −8.79 to −5.63; *p* < 0.00001; *I* ^2^ = 96%) and waist circumference (MD −14.27 cm; 95% CI −15.43 to −13.10; *p* < 0.00001; *I* ^2^ = 23%) versus placebo. No significant subgroup differences were observed for BMI (*p* = 0.07) or waist circumference (*p* = 0.61) (Figure S1A,B). Higher proportions of tirzepatide‐treated participants achieved weight loss thresholds of ≥ 5% (RR 3.69), ≥ 10% (RR 7.04) and ≥ 15% (RR 14.29) versus placebo, with no significant ethnic subgroup interactions across all categories (*p* ≥ 0.05) (Figure S2A–C).

### 3.7 Sensitivity Analysis

The sensitivity analysis restricted to the trials with fixed‐dose tirzepatide 15 mg arms (SURMOUNT‐1, SURMOUNT‐CN, SURMOUNT‐J) yielded results that were directionally consistent with the primary analysis for percentage weight change (Figure S3A). This supports the robustness of the primary findings with respect to the heterogeneity in dosing strategy introduced by including MTD arms. In contrast, the previously significant subgroup difference in absolute weight loss was attenuated and no longer statistically significant in the fixed‐dose sensitivity analysis. This indicates that the larger kilogram loss observed in predominantly non‐Asian trials in the primary analysis was at least partly driven by dosing heterogeneity and selection into MTD arms, rather than by ethnicity‐specific effects (Figure S3B).

### 3.8 Adverse Events

Tirzepatide 15 mg/MTD significantly increased the risk of at least one AE (RR 1.11; 95% CI 1.07–1.16), gastrointestinal AEs including nausea (RR 3.18), diarrhoea (RR 3.05), vomiting (RR 6.33), abdominal pain (RR 2.23), eructation (RR 7.85) and decreased appetite (RR 2.32) versus placebo. It also increased trial drug discontinuation due to AEs (RR 2.12) and severe/serious gastrointestinal AEs (RR 3.48). No increased risk of serious AEs (RR 0.90) or pancreatitis (RR 1.61) was observed. For all AE outcomes, chi‐square tests for subgroup interaction showed no statistically significant differences between Asian and predominantly non‐Asian trials, with overlapping confidence intervals. Numerically higher RRs for nausea and diarrhoea were observed in Asian trials, and for vomiting in predominantly non‐Asian trials, but these differences did not reach statistical significance and should be interpreted as hypothesis‐generating (Table 2).

**TABLE 2:** Risk ratios for the adverse events in tirzepatide 15 mg/maximum tolerated dose versus placebo.

Outcome variables	Group	Number of RCTs (number of study subjects)	RR [95% CI]	*p*	*I* ^2^ (%)	*p* for subgroup differences
Subjects with ≥ 1 treatment‐emergent adverse events	All subjects	6 (2611)	1.11 [1.07, 1.16]	< 0.00001	0	
	Asian	2 (292)	1.15 [1.02, 1.29]	0.03	19	0.60
	Predominantly non‐Asian	4 (2319)	1.11 [1.06, 1.16]	< 0.00001	0	
Serious adverse events	All subjects	6 (2611)	0.90 [0.67, 1.21]	0.47	0	
	Asian	2 (292)	1.15 [0.53, 2.49]	0.72	0	0.50
	Predominantly non‐Asian	4 (2319)	0.86 [0.62, 1.21]	0.39	5	
Adverse events leading to discontinuation of trial drug	All subjects	6 (2611)	2.12 [1.06, 4.23]	0.03	59	
	Asian	2 (292)	1.96 [0.75, 5.11]	0.17	0	0.95
	Predominantly non‐Asian	4 (2319)	2.05 [0.78, 5.39]	0.15	75	
Severe or serious gastrointestinal adverse events	All subjects	6 (2611)	3.48 [1.93, 6.29]	< 0.0001	0	
	Asian	2 (292)	3.89 [0.45, 33.91]	0.22	NA	0.92
	Predominantly non‐Asian	4 (2319)	3.45 [1.87, 6.39]	< 0.0001	0	
Nausea	All subjects	6 (2611)	3.18 [2.65, 3.82]	< 0.00001	0	
	Asian	2 (292)	5.69 [2.65, 12.26]	< 0.00001	0	0.12
	Predominantly non‐Asian	4 (2319)	3.18 [2.65, 3.82]	< 0.00001	0	
Diarrhoea	All subjects	6 (2611)	3.05 [2.49, 3.74]	< 0.00001	0	
	Asian	2 (292)	4.10 [1.87, 8.98]	0.0004	16	0.43
	Predominantly non‐Asian	4 (2319)	2.96 [2.39, 3.66]	< 0.00001	0	
Vomiting	All subjects	6 (2611)	6.33 [4.24, 9.44]	< 0.00001	0	
	Asian	2 (292)	3.68 [1.54, 8.81]	0.003	0	0.17
	Predominantly non‐Asian	4 (2319)	7.31 [4.66, 11.47]	< 0.00001	0	
Abdominal pain	All subjects	6 (2611)	2.23 [1.33, 3.74]	0.002	26	
	Asian	2 (292)	2.27 [0.60, 8.58]	0.23	0	0.99
	Predominantly non‐Asian	4 (2319)	2.25 [1.23, 4.12]	0.009	42	
Eructation	All subjects	6 (2611)	7.85 [4.00, 15.38]	< 0.00001	0	
	Asian	2 (292)	6.60 [0.81, 53.55]	0.08	0	0.86
	Predominantly non‐Asian	4 (2319)	8.01 [3.93, 16.30]	< 0.00001	0	
Decreased appetite	All subjects	6 (2611)	2.32 [1.67, 3.21]	< 0.00001	0	
	Asian	2 (292)	2.68 [1.33, 5.44]	0.006	0	0.65
	Predominantly non‐Asian	4 (2319)	2.32 [1.67, 3.21]	< 0.00001	0	
Adjudicated pancreatitis	All subjects	6 (2611)	1.61 [0.31, 8.32]	0.57	0	
	Asian	2 (292)	Not estimable	NA	NA	NA
	Predominantly non‐Asian	4 (2319)	1.61 [0.31, 8.32]	0.57	0	

Abbreviations: CI, confidence interval; NA, not applicable; RR, risk ratio.

## 4 Discussion

This systematic review and meta‐analysis of the SURMOUNT trials (*N* = 4022) found that tirzepatide 15 mg/MTD produced large, clinically meaningful reductions in body weight and adiposity indices among adults with obesity without diabetes, with broadly comparable percentage weight loss across Asian and predominantly non‐Asian trial populations. The absence of a statistically significant subgroup interaction for percentage weight change (*p* for interaction = 0.94) should not be interpreted as evidence of equivalence, because the Asian subgroup comprised only two trials and 148 tirzepatide‐treated participants, leaving the analysis underpowered to detect modest but clinically relevant differences. These findings are broadly consistent with prior ethnicity‐focused meta‐analytic data from tirzepatide trials in type 2 diabetes, which reported largely comparable relative weight loss across Asian and non‐Asian groups, even though some glycemic endpoints favoured non‐Asian participants [16].

The greater absolute weight loss observed in predominantly non‐Asian trials warrants cautious interpretation. Trial‐level meta‐regression identified trial duration as a key moderator of both percentage and absolute weight change, with longer studies showing larger reductions and suggested that higher baseline BMI and body weight may further amplify absolute kilogram loss. However, the relatively high *R* ^2^ values observed in some meta‐regression models (approximately 50%–65%) may reflect overfitting given the limited number of trials and should not be interpreted as precise predictive estimates. Taken together, these findings indicate that between‐trial differences in baseline phenotype and exposure duration account for much of the greater absolute weight loss in non‐Asian trials, rather than indicating an intrinsic ethnicity‐specific pharmacodynamic effect. A pre‐specified sensitivity analysis offers further context. When restricted to trials using fixed‐dose 15 mg tirzepatide (SURMOUNT‐1, SURMOUNT‐CN, SURMOUNT‐J), estimates of percentage weight change remained directionally consistent with the primary analysis, supporting broadly similar relative efficacy across Asian and predominantly non‐Asian trial populations, whereas the previously significant subgroup difference in absolute weight loss was attenuated and no longer statistically significant. In this fixed‐dose framework, the excess absolute weight loss in non‐Asian trials appears partly attributable to dosing heterogeneity and selection into maximum‐tolerated‐dose arms. Asian trials contributed only fixed 15 mg data, whereas some non‐Asian trials included MTD arms in which not all participants reached 15 mg, creating a structural asymmetry. Because escalation to MTD in SURMOUNT‐3 and SURMOUNT‐OSA depended on individual tolerability, participants who did not attain 15 mg are likely to have systematically different efficacy and safety profiles, introducing non‐random missingness and selection bias that further limit trial‐level ethnic inferences.

The overall adverse‐event profile in this analysis was consistent with the established safety profile of tirzepatide in obesity and type 2 diabetes trials [8, 23]. Tirzepatide 15 mg/MTD significantly increased the risk of any treatment‐emergent adverse event and of gastrointestinal adverse events (including nausea, diarrhoea, vomiting, abdominal pain, eructation and decreased appetite) and increased discontinuations due to adverse events and severe/serious gastrointestinal adverse events compared with placebo. However, there was no evidence of increased serious adverse events overall or of pancreatitis and no statistically significant subgroup interaction was observed for any safety outcome, with confidence intervals overlapping between Asian and non‐Asian trials. Numerically higher risk ratios for nausea and diarrhoea were observed in Asian trials, whereas vomiting appeared numerically higher in predominantly non‐Asian trials. These patterns echo prior reports suggesting that Asian populations may exhibit greater gastrointestinal sensitivity to incretin‐based therapies [24]. However, the wide confidence intervals and non‐significant interaction tests mean that these findings should be considered hypothesis‐generating rather than definitive.

The descriptive 10 mg data provide clinical insights, particularly for Asian populations with lower body weight and tolerability concerns that influence dose selection. In SURMOUNT‐1, SURMOUNT‐CN and SURMOUNT‐J, tirzepatide 10 mg significantly reduced body weight, BMI and waist circumference, with effects that were directionally similar to 15 mg but smaller, as expected for a lower dose. 10 mg safety data showed the anticipated gastrointestinal profile—higher rates of nausea, diarrhoea, vomiting and decreased appetite compared with placebo—without any signal of excess serious adverse events or pancreatitis, including in the exclusively Asian SURMOUNT‐CN and SURMOUNT‐J populations. These descriptive findings do not permit a formal ethnic dose–response comparison due to the small number of 10‐mg trials and the mixed‐ethnicity makeup of SURMOUNT‐1, but they suggest a reasonable hypothesis that some Asian adults might find that doses below 15 mg provide a good balance between effectiveness and gastrointestinal tolerability.

These findings support several practical considerations for tirzepatide prescribing in diverse populations. First, comparable relative weight loss across Asian and non‐Asian trial populations, despite substantially lower baseline BMI in Asian trials (32–34 vs. 36–40 kg/m^2^), reinforces the appropriateness of using lower BMI thresholds (≥ 25 or ≥ 27.5 kg/m^2^) when considering tirzepatide initiation in Asian adults, consistent with regional obesity guidelines. Second, clinicians should frame treatment expectations and monitor response primarily by percentage weight loss rather than by absolute kilograms, because baseline body weight substantially influences the latter but not the former. Third, the descriptive 10 mg data suggest that dose individualisation may be particularly valuable in Asian populations, where intermediate doses appear to provide clinically meaningful weight reduction and may have a more favourable gastrointestinal tolerability profile than 15 mg, though formal dose–response comparisons by ethnicity are lacking. Fourth, the broadly similar safety profile across ethnic subgroups supports the use of tirzepatide in diverse populations, though ongoing vigilance for gastrointestinal adverse events remains essential, with possible heightened attention to Asian patients given numerically higher rates of nausea and diarrhoea. Finally, these exploratory findings underscore the importance of culturally contextualised obesity care that integrates medication management with locally appropriate dietary patterns, family structures and healthcare system realities, particularly in Asian settings where obesity pharmacotherapy remains underutilised despite a rising disease burden.

### 4.1 Strengths and Limitations of the Study

The present study has several strengths and important limitations. Strengths include the exclusive focus on phase 3, randomised, placebo‐controlled SURMOUNT trials in adults with obesity without diabetes; the use of a pre‐specified, trial‐level ethnic classification to maximise contrast between Asian and predominantly non‐Asian populations; a comprehensive assessment of efficacy and safety endpoints; the incorporation of exploratory meta‐regression to evaluate whether trial‐level baseline body size, age, sex distribution and duration accounted for between‐trial heterogeneity; and a fixed‐dose sensitivity analysis to address concerns about dosing‐strategy heterogeneity. However, the ethnic comparison is indirect and ecological: no SURMOUNT trial randomised participants by ethnicity, so observed similarities or differences cannot be causally attributed to ethnicity per se and may reflect between‐trial differences in eligibility criteria, BMI thresholds, background lifestyle counselling, dosing regimens and healthcare settings. The Asian evidence base is limited to two East Asian trials (SURMOUNT‐CN and SURMOUNT‐J) and 148 tirzepatide‐treated participants, constraining generalisability to South Asian, Southeast Asian and other Asian populations. SURMOUNT‐OSA(1) and SURMOUNT‐OSA(2) were classified as predominantly non‐Asian because ethnicity‐stratified data were unavailable, despite including up to 20% Asian participants, potentially introducing misclassification bias. Dose–response information remains incomplete because the 5 and 10 mg arms were excluded from the primary meta‐analysis, and all trials used placebo rather than active comparators, precluding ethnic subgroup comparisons against other anti‐obesity medications. Finally, follow‐up ranged from 52 to 72 weeks; longer‐term data beyond 72 weeks are lacking, and the meta‐regression and sensitivity analyses are underpowered given the small number of available trials.

## 5 Conclusions

Tirzepatide 15 mg/MTD produced large, clinically meaningful weight loss in adults with obesity without diabetes, with broadly similar relative percentage efficacy across Asian and predominantly non‐Asian SURMOUNT populations. The greater absolute weight loss in non‐Asian trials appears to be mainly driven by higher baseline body weight and BMI, and longer trial duration, as supported by meta‐regression and by the attenuation of subgroup differences in sensitivity analyses for fixed‐dose 15 mg. These exploratory, hypothesis‐generating findings are constrained by a small, geographically restricted Asian evidence base and an indirect, underpowered ethnic comparison. Dedicated, prospective, ethnicity‐stratified trials with adequate power are needed to define ethnicity‐related differences in tirzepatide response and to inform personalised, ethnicity‐conscious obesity management.

## Author Contributions

**Joseph M. Pappachan:** validation, writing – review and editing, resources, supervision, methodology. **Kunal Mahajan:** writing – original draft, methodology, resources. **Lakshmi Nagendra:** investigation, data curation, resources, software, writing – review and editing. **Deep Dutta:** conceptualization, validation, project administration, resources, writing – review and editing. **A. B. M. Kamrul‐Hasan:** conceptualization, methodology, software, visualization, project administration, writing – original draft, supervision, formal analysis. **Ibrahim Khalil:** investigation, data curation, software, resources, visualization, writing – review and editing, formal analysis.

## Funding

The authors have nothing to report.

## Conflicts of Interest

The authors declare no conflicts of interest.

## Supporting information


**Table S1:** Search strings for the databases.
**Table S2:** Descriptive summary of efficacy outcomes for tirzepatide 10 mg in SURMOUNT‐1, SURMOUNT‐CN and SURMOUNT‐J.
**Table S3:** Descriptive summary of safety outcomes for tirzepatide 10 mg in SURMOUNT‐1, SURMOUNT‐CN and SURMOUNT‐J.
**Table S4:** Univariable meta‐regression of trial‐level predictors for percentage body weight change with tirzepatide versus placebo (six studies).
**Table S5:** Univariable meta‐regression of trial‐level predictors for absolute body weight change with tirzepatide versus placebo (four studies).
**Figure S1:** Forest plot displaying. (A) Changes in body mass index from baseline in tirzepatide versus placebo groups; (B) Changes in waist circumference from baseline in tirzepatide versus placebo groups.
**Figure S2:** Forest plot displaying. (A) Proportion of study subjects achieving weight reduction of 5% or more from baseline in the tirzepatide versus placebo groups; (B) Proportion of study subjects achieving weight reduction of 10% or more from baseline in the tirzepatide versus placebo groups; (C) Proportion of study subjects achieving weight reduction of 15% or more from baseline in the tirzepatide versus placebo groups.
**Figure S3:** Sensitivity analysis restricted to the fixed‐dose tirzepatide 15 mg arms (SURMOUNT‐1, SURMOUNT‐CN, SURMOUNT‐J) for A. Percent change in body weight (%) and B. Absolute change in body weight (kg).

## Data Availability

All datasets created or examined in this research are included in this published article and its Supporting Information.
