# Supplementary material for: Comparative Efficacy and Safety of Tirzepatide in Asian and Non‐Asian Adults With Obesity Without Diabetes: A Systematic Review and Meta‐Analysis
Source: Endocrinol Diabetes Metab. 2026 Jul 22;9(4):e70291. doi: 10.1002/edm2.70291 (PMC13392206; doi:10.1002/edm2.70291)
Supplement: Supplementary file 1 — Table S1: Search strings for the databases. Table S2: Descriptive summary of efficacy outcomes for tirzepatide 10 mg in SURMOUNT‐1, SURMOUNT‐CN and SURMOUNT‐J. Table S3: Descriptive summary of safety outcomes for tirzepatide 10 mg in SURMOUNT‐1, SURMOUNT‐CN and SURMOUNT‐J. Table S4: Univariable meta‐regression of trial‐level predictors for percentage body weight change with tirzepatide versus placebo (six studies). Table S5: Univariable meta‐regression of trial‐level predictors for absolute body weight change with tirzepatide versus placebo (four studies). Figure S1: Forest plot displaying. (A) Changes in body mass index from baseline in tirzepatide versus placebo groups; (B) Changes in waist circumference from baseline in tirzepatide versus placebo groups. Figure S2: Forest plot displaying. (A) Proportion of study subjects achieving weight reduction of 5% or more from baseline in the tirzepatide versus placebo groups; (B) Proportion of study subjects achieving weight reduction of 10% or more from baseline in the tirzepatide versus placebo groups; (C) Proportion of study subjects achieving weight reduction of 15% or more from baseline in the tirzepatide versus placebo groups. Figure S3: Sensitivity analysis restricted to the fixed‐dose tirzepatide 15 mg arms (SURMOUNT‐1, SURMOUNT‐CN, SURMOUNT‐J) for A. Percent change in body weight (%) and B. Absolute change in body weight (kg). [file EDM2-9-e70291-s001.docx]

**Table S1. Search strings for the databases**

| PubMed | (tirzepatide[tiab] OR tirzepatide[Mesh] OR LY3298176[tiab])  AND  (obesity[Mesh] OR obes*[tiab] OR overweight[tiab] OR "weight loss"[tiab] OR "weight reduction"[tiab])  AND  ("randomized controlled trial"[pt] OR "clinical trial"[pt] OR randomized[tiab] OR randomised[tiab]) |
| --- | --- |
| Scopus | (TITLE-ABS-KEY(tirzepatide OR LY3298176))  AND  (TITLE-ABS-KEY(obes* OR overweight OR "weight loss" OR "weight reduction"))  AND  (TITLE-ABS-KEY("randomized controlled trial" OR "randomised controlled trial" OR randomized OR randomised OR "clinical trial")) |
| Web of Science | TS=((tirzepatide OR LY3298176) AND (obes* OR overweight OR "weight loss" OR "weight reduction") AND ("randomized controlled trial" OR "randomised controlled trial" OR randomized OR randomised OR "clinical trial")) |

**Table S2. Descriptive summary of efficacy outcomes for tirzepatide 10 mg in SURMOUNT-1, SURMOUNT-CN, and SURMOUNT-J**

| **Outcome** | **SURMOUNT-1** | | **SURMOUNT-CN** | | **SURMOUNT-J** | |
| --- | --- | --- | --- | --- | --- | --- |
|  | **Tirzepatide 10 mg (n=636)** | **Placebo (n=643)** | **Tirzepatide 10 mg (n=70)** | **Placebo (n=69)** | **Tirzepatide 10 mg (n=73)** | **Placebo (n=75)** |
| Mean % change in body weight, % | −19.5 | −3.1 | −13.6 | −2.3 | −17.8 | −1.7 |
| Mean absolute body weight change, kg | −23.2 | −2.4 | −12.3 | −2.1 | −16.0 | −1.5 |
| Participants achieving ≥5% weight reduction, % | 88.9 | 34.5 | 87.7 | 29.3 | 94 | 20 |
| Participants achieving ≥10% weight reduction, % | 78.1 | 18.8 | 64.1 | 14.5 | 86 | 4 |
| Participants achieving ≥15% weight reduction, % | 66.6 | 8.8 | 46.4 | 2.9 | 63 | 1 |
| Mean change in body mass index, kg/m^2^ | −8.1 | −0.9 | −4.4 | −0.7 | −5.8 | −0.6 |
| Mean change in waist circumference, cm | −17.7 | −4.0 | −11.4 | −2.6 | −12.7 | −1.3 |

**Table S3. Descriptive summary of safety outcomes for tirzepatide 10 mg in SURMOUNT-1, SURMOUNT-CN, and SURMOUNT-J**

| **Adverse Event** | **SURMOUNT-1** | | **SURMOUNT-CN** | | **SURMOUNT-J** | |
| --- | --- | --- | --- | --- | --- | --- |
|  | **Tirzepatide 10 mg (n=636)** | **Placebo (n=643)** | **Tirzepatide 10 mg (n=70)** | **Placebo (n=69)** | **Tirzepatide 10 mg (n=73)** | **Placebo (n=75)** |
| ≥1 treatment-emergent adverse event | 520 (81.8%) | 463 (72.0%) | 67 (95.7%) | 57 (82.6%) | 61 (84%) | 52 (69%) |
| Serious adverse events | 44 (6.9%) | 44 (6.8%) | 3 (4.3%) | 6 (8.7%) | 8 (11%) | 5 (7%) |
| Adverse events leading to discontinuation of trial drug | 45 (7.1%) | 17 (2.6%) | 2 (2.9%) | 1 (1.4%) | 7 (10%) | 5 (7%) |
| Severe or serious gastrointestinal adverse events | 20 (3.1%) | 7 (1.1%) | 2 (2.9%) | 1 (1.4%) | NR | NR |
| Nausea | 212 (33.3%) | 61 (9.5%) | 21 (30.0%) | 4 (5.8%) | 10 (14%) | 3 (4%) |
| Diarrhoea | 135 (21.2%) | 47 (7.3%) | 28 (40.0%) | 6 (8.7%) | 9 (12%) | 3 (4%) |
| Vomiting | 68 (10.7%) | 11 (1.7%) | 8 (11.4%) | 3 (4.3%) | 5 (7%) | 3 (4%) |
| Abdominal pain | 34 (5.3) | 21 (3.3%) | 4 (7.14%) | 2 (2.9%) | 2 (2.74%) | 1 (1.33%) |
| Eructation | 33 (5.2%) | 4 (0.6%) | NR | NR | 1 (1.37%) | 0 |
| Decreased appetite | 73 (11.5%) | 21 (3.3%) | 19 (27.1%) | 8 (11.6%) | 9 (12%) | 1 (1%) |
| Adjudicated pancreatitis | 1 (0.2%) | 1 (0.2%) | 0 | 0 | 0 | 0 |

NR, not reported

**Table S4. Univariable meta-regression of trial-level predictors for percentage body weight change with tirzepatide versus placebo (six studies)**

| **Moderator** | **Beta coefficient** | **95% CI Lower** | **95% CI Upper** | **p-value** | **SE** | **Z** | **R^2^ (%)** |
| --- | --- | --- | --- | --- | --- | --- | --- |
| Mean Baseline Body Weight (kg) | 0.0728 | -0.1150 | 0.2607 | 0.4471 | 0.0958 | 0.7602 | 0.00 |
| Mean Baseline BMI (kg/m^2^) | 0.1562 | -0.5770 | 0.8895 | 0.6762 | 0.3741 | 0.4176 | 0.00 |
| Mean Baseline Age (years) | -0.2198 | -0.5930 | 0.1534 | 0.2483 | 0.1904 | -1.1545 | 3.77 |
| Percentage of female | -0.0398 | -0.1594 | 0.0798 | 0.5139 | 0.0610 | -0.6528 | 0.00 |
| Trial Duration (weeks) | **-0.1643** | **-0.2937** | **-0.0350** | **0.0128** | **0.0660** | **-2.4905** | **64.48** |

**Table S5: Univariable meta-regression of trial-level predictors for absolute body weight change with tirzepatide versus placebo (four studies)**

| **Moderator** | **Beta coefficient** | **95% CI Lower** | **95% CI Upper** | **p-value** | **SE** | **Z** | **R^2^ (%)** |
| --- | --- | --- | --- | --- | --- | --- | --- |
| Mean Baseline Body Weight (kg) | -0.4983 | -1.0237 | 0.0270 | 0.0630 | 0.2680 | -1.8592 | 47.79 |
| Mean Baseline BMI (kg/m^2^) | -1.3824 | -2.8010 | 0.0362 | 0.0561 | 0.7238 | -1.9099 | 49.48 |
| Mean Baseline Age (years) | -0.4362 | -1.2633 | 0.3910 | 0.3014 | 0.4220 | -1.0335 | 1.55 |
| Percentage of female | -0.2546 | -0.6601 | 0.1508 | 0.2184 | 0.2069 | -1.2309 | 15.26 |
| Trial Duration (weeks) | -0.4065 | -0.7387 | -0.0744 | **0.0165** | 0.1695 | -2.3987 | 65.58 |

**Figure S1. Forest plot displaying A. Changes in body mass index from baseline in Tirzepatide vs. Placebo groups; B. Changes in waist circumference from baseline in Tirzepatide vs. Placebo groups.**


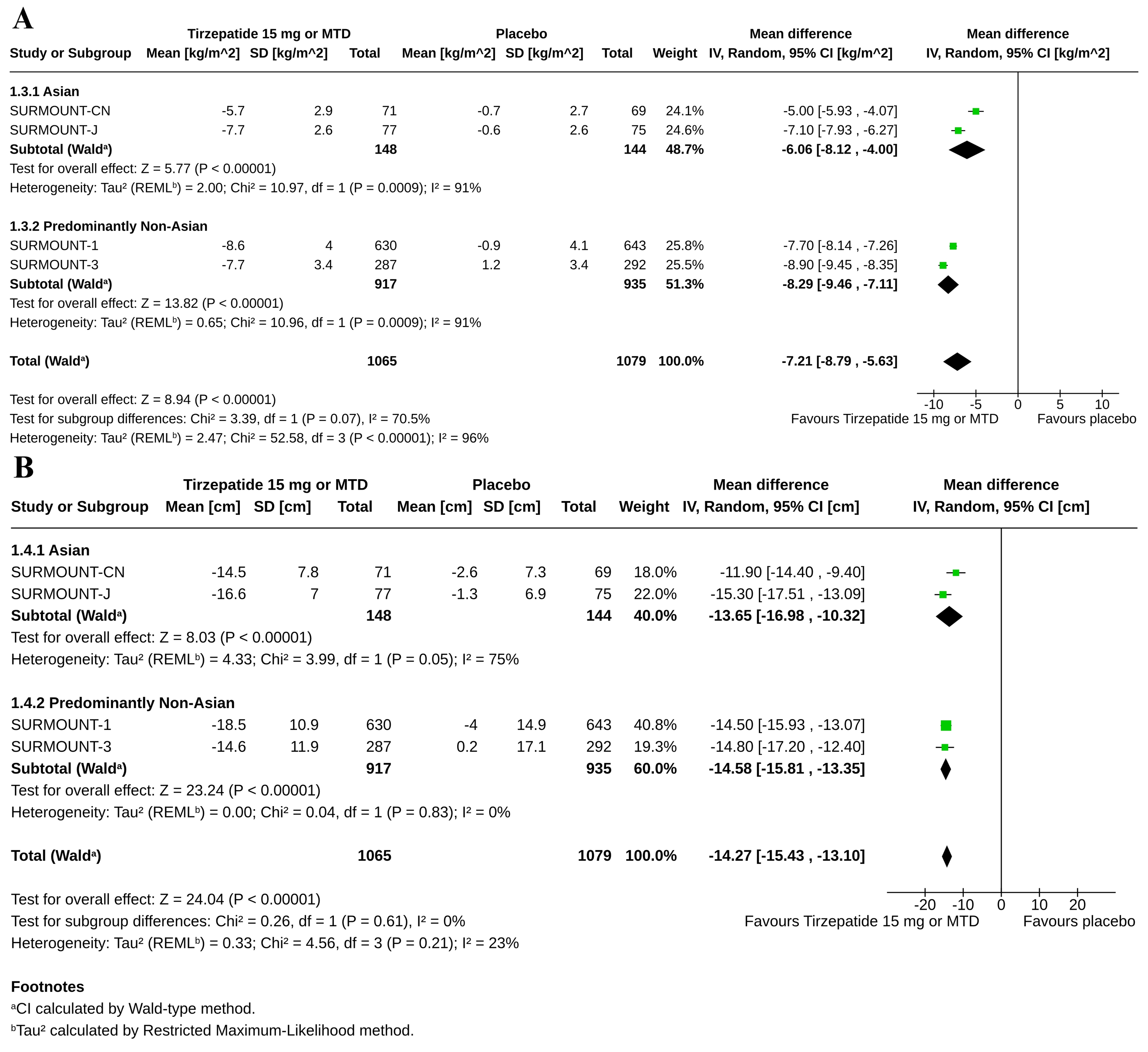


**Figure S2. Forest plot displaying A. Proportion of study subjects achieving weight reduction of 5% or more from baseline in the Tirzepatide vs. Placebo groups; B. Proportion of study subjects achieving weight reduction of 10% or more from baseline in the Tirzepatide vs. Placebo groups; C. Proportion of study subjects achieving weight reduction of 15% or more from baseline in the Tirzepatide vs. Placebo groups.**

**
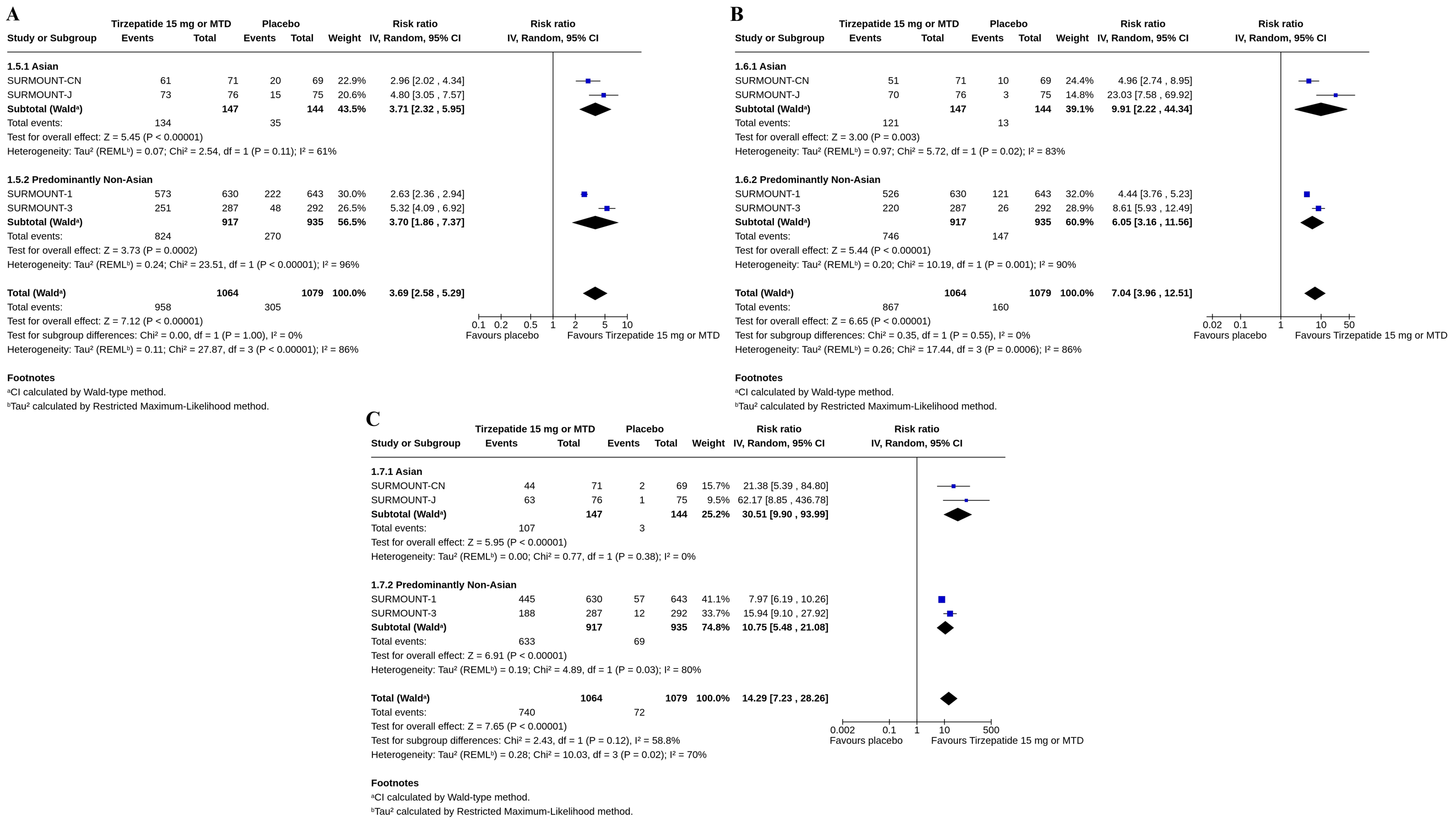
**

**Figure S3. Sensitivity analysis restricted to the fixed-dose tirzepatide 15 mg arms (SURMOUNT-1, SURMOUNT-CN, SURMOUNT-J) for A. Percent change in body weight (%), and B. Absolute change in body weight (kg)**

**
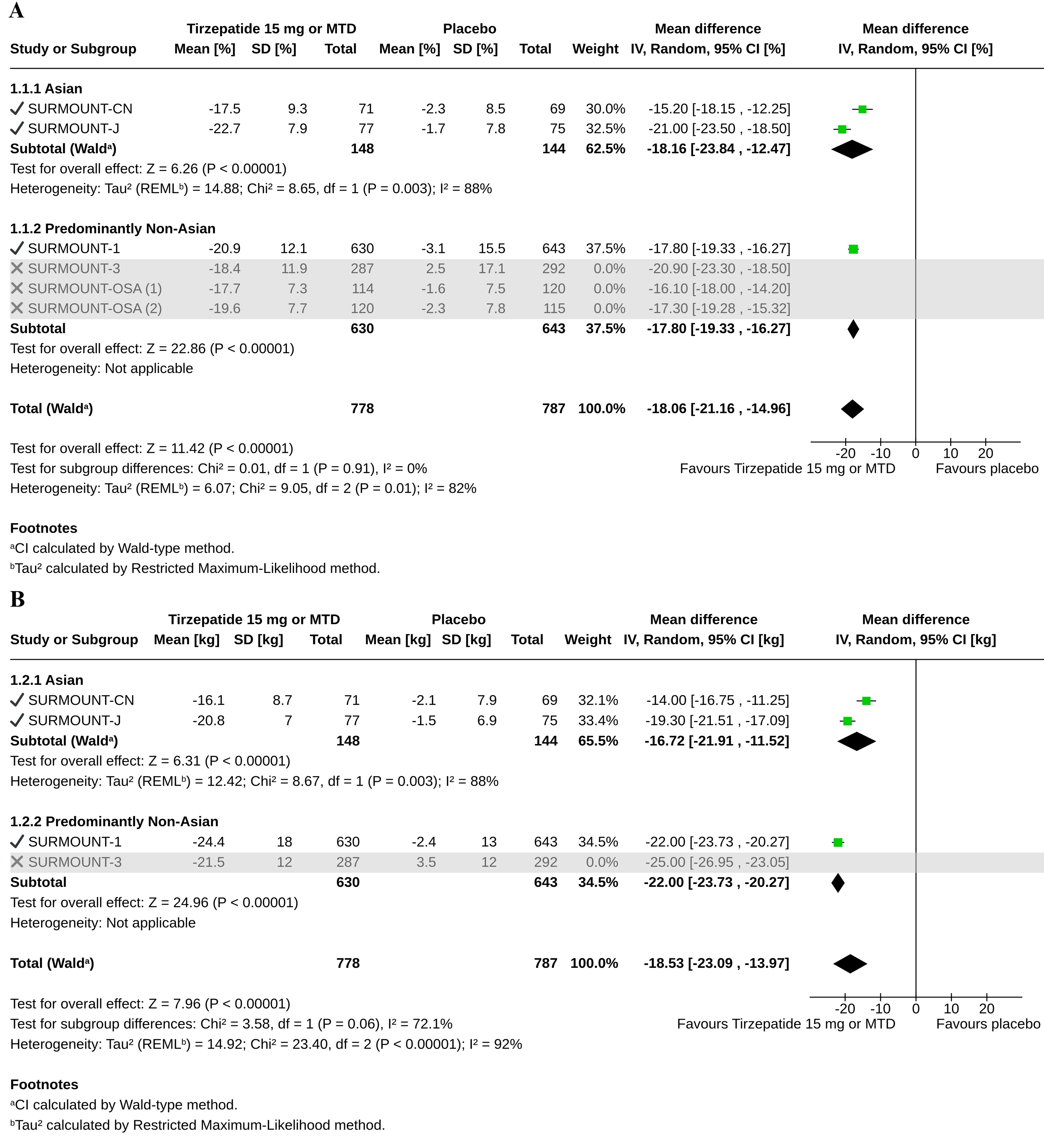
**
